# Exposure to Parents’ Negative Emotions as a Developmental Pathway to the Family Aggregation of Depression and Anxiety in the First Year of Life

**DOI:** 10.1007/s10567-017-0240-7

**Published:** 2017-05-20

**Authors:** Evin Aktar, Susan M. Bögels

**Affiliations:** 1 Clinical Psychology Unit, Leiden University, Wassenaarseweg 52, 2333 AK Leiden, The Netherlands; 2 Research Institute of Child Development and Education, University of Amsterdam, Nieuwe Achtergracht 127, 1001 NG Amsterdam, The Netherlands

**Keywords:** Environmental exposure, Parental emotion, Depression, Anxiety, Temperament, Infancy

## Abstract

Depression and anxiety load in families. In the present study, we focus on exposure to parental negative emotions in first postnatal year as a developmental pathway to early parent-to-child transmission of depression and anxiety. We provide an overview of the little research available on the links between infants’ exposure to negative emotion and infants’ emotional development in this developmentally sensitive period, and highlight priorities for future research. To address continuity between normative and maladaptive development, we discuss exposure to parental negative emotions in infants of parents with as well as without depression and/or anxiety diagnoses. We focus on infants’ emotional expressions in everyday parent–infant interactions, and on infants’ attention to negative facial expressions as early indices of emotional development. Available evidence suggests that infants’ emotional expressions echo parents’ expressions and reactions in everyday interactions. In turn, infants exposed more to negative emotions from the parent seem to attend less to negative emotions in others’ facial expressions. The links between exposure to parental negative emotion and development hold similarly in infants of parents with and without depression and/or anxiety diagnoses. Given its potential links to infants’ emotional development, and to later psychological outcomes in children of parents with depression and anxiety, we conclude that early exposure to parental negative emotions is an important developmental mechanism that awaits further research. Longitudinal designs that incorporate the study of early exposure to parents’ negative emotion, socio-emotional development in infancy, and later psychological functioning while considering other genetic and biological vulnerabilities should be prioritized in future research.

## Introduction

Depression and anxiety disorders are among the most prevalent psychopathology in children (Kashani and Orvaschel 1990) and adults (Alonso et al. 2004; Jacobi et al. 2004; Kessler et al. 2005). Depression and anxiety aggregate in families, in other words parents’ depression and anxiety disorders constitute risk for depression and anxiety in the offspring (Beardslee et al. 2011; Beidel and Turner 1997; Turner et al. 1987). Infants born to depressed or anxious parents not only inherit a genetic vulnerability that predisposes them to depression and anxiety, but they also grow in socio-emotional environments marked by alterations in parents’ emotional expressions (Eley et al. 2015; Hettema et al. 2001; Nivard et al. 2015). Despite the significant family loading, some children with depressed/anxious parents never develop depression/anxiety. For those who do, there is a big variability in the outcomes. A developmental psychopathology perspective on the intergenerational transmission of depression and anxiety embraces the diversity in the developmental pathways to depression and anxiety (Cicchetti and Toth 1998; Dadds and Vasey 2001). Differently from previous work that addressed this diversity (Creswell and Waite 2015; Goodman and Gotlib 1999; Murray et al. 2009), the current review specifically focuses on infants’ exposure to depressed/anxious parents’ negative emotions as an early developmental pathway to the intergenerational transmission.

From a developmental psychopathology perspective, both adaptation and maladaptation arise from complex and dynamic transactions across psychological, biological, and social mechanisms operating at the intrapersonal and the interpersonal levels (Cicchetti and Dawson 2002; Sroufe 1990; Sroufe and Rutter 1984). It is therefore important to keep in mind that exposure effects that constitute the main scope of the current review are only one among many developmental mechanisms that may contribute to parent-to-child transmission of depression and anxiety. Isolated consideration of exposure effects can therefore not be sufficient to explain the observed variation in the child outcomes, or in the emergence of psychopathology in the offspring of depressed and anxious parents. Understanding how multiple transactions between infants’ exposure to parental depression and anxiety and other characteristics of the parent, the child and the environment dynamically determine the outcomes in the offspring remains essential (Goodman and Gotlib 1999; Murray et al. 2009).

The developmental models of parent-to-child transmission of depression (Goodman and Gotlib 1999) and anxiety (Murray et al. 2009) propose that children’s repeated exposure to parents’ depressed and anxious moods is a potential mechanism that contributes to risk for the development of psychopathology. Goodman and Gotlib (1999) proposed that this mechanism consists of 5 inter-related components in the case of maternal depression. Here, we briefly summarize these components and extend them to anxiety disorders and to fathers. (1) Depression and anxiety in parents are defined by heightened duration, frequency and intensity of negative emotions, moods and behaviors (see American Psychiatric Association (APA) 2013). Depressed parents experience more flat and negative affect (sadness, irritability, anger, and guilt) in their interactions in everyday life. Anxious parents experience excessive fear, anxiety and worry either in response to certain specific situations or stimuli or in general (in the case of generalized anxiety disorder). (2) Due to their negative emotions/affect, parents with depression and/or anxiety may not be able to provide the optimal interpersonal environment for their children. (3) The lack of an optimal interpersonal environment will adversely affect children’s socio-emotional development. (4) Because of these adverse effects, children will acquire negative behaviors and moods that look like negative behaviors and moods of depressed parents. Finally, (5) acquisition of these negative behaviors and moods will put infants at risk for the development of anxiety and depression.

How do exposure effects dynamically change throughout development? Are there developmentally sensitive periods where the adverse effects of exposure to maternal depression/anxiety are especially pronounced? Is there a time in development where the offspring becomes immune to exposure to parents’ negative moods? To seek for answers to these questions, it is necessary to place exposure effects in a developmental framework. Building on the available evidence, the current study proposes that the first postnatal year constitutes a developmentally sensitive period for the effects of exposure to parental depression and anxiety.

Why is it important to specifically focus on the first year of life to discuss exposure effects in the intergenerational transmission of depression and anxiety? Higher prevalence of depression and anxiety in the postnatal years reveals that early years of parenthood may be a vulnerable period for the depression and anxiety in parents (Matthey et al. 2003; O’Hara and Swain 1996; Ross and McLean 2006). In turn, the rapid, experience-dependent development of emotional brain systems at this period seems to make infants particularly vulnerable to the effects of exposure to parental depression and anxiety (Leppänen 2011; Leppänen and Nelson 2009). Leppänen and colleagues suggest that infants’ exposure to parents’ expressions of emotion during daily parent–infant interactions plays an essential role in the neural fine-tuning of infants’ emotional brain systems in typical development (Leppänen 2011; Leppänen and Nelson 2009). Findings on face processing in typically developing infants illustrate the important role that exposure to primary caregivers’ faces may have on shaping attention in the first year of life: Because of being predominantly exposed to mothers’ faces, infants whose mothers are their primary caregivers show enhanced attention to mothers’ (vs. strangers’) neutral faces (3-to-6 months, De Haan and Nelson 1997, 1999; Montague and Walker-Andrews 2002). Infants’ exposure to mothers’ faces seems to also shape their attention to strangers’ faces: 3-month-old infants look longer to stranger faces if the gender of the stranger matches the gender of their primary caregiver (Quinn and Slater 2003; Quinn et al. 2002). Thus, exposure to parents’ faces in typical development tunes infants’ attention into the caregiving parents’ faces, ensuring enhanced processing of the most relevant and most frequently encountered stimuli in the environment.

Given this specific importance of environmental exposure for the development of emotional brain systems, Leppänen predicts that the influence of an atypical emotional environment provided by depressed and/or anxious parents in the early years would be *‘especially detrimental’* for later development of emotion processing abilities (Leppänen 2011, p. 185). In a parallel vein, Goodman and Gotlib (1999) suggest that the time of exposure to parents’ depression and anxiety determines the strength of influence on child outcomes, with early years of life constituting the most vulnerable period.

But what are the effects of parental depression and/or anxiety in the first year of life on the offspring’s current and later functioning? Evidence on the effects of parental depression and anxiety in the postnatal year on later psychopathology in childhood and beyond has only started to accumulate. Research in this area is limited by certain  methodological issues (such as reliance on mothers’ report of infants’ functioning, and lack of control for prenatal or concurrent depression). Nevertheless available evidence provides preliminary support for the idea that parents’ depression and anxiety may have prolonged effects on offsprings’ functioning and psychopathology. For example, longitudinal links were shown between mothers’ depressive symptoms in the first year and child behavioral and/or emotional problems at 2 (Avan et al. 2010), and 5 years of age (Murray et al. 1999). Evidence also shows a significant increase in the risk of overall psychopathology at the age of 11 in children of postnatally depressed mothers (Pawlby et al. 2008), and an increase in depression and anxiety diagnoses at the age of 13 (Halligan et al. 2007) and of 16 (Murray et al. 2011). Likewise, paternal depression was found to be linked to more behavioral and emotional problems in later childhood (Ramchandani et al. 2008; Ramchandani and Psychogiou 2009). Moreover, it seems that paternal depression may have effects on child outcomes that are specific and independent of mothers’ depression (Ramchandani et al. 2005). The available evidence on  the effects of postnatal maternal anxiety reveals similar links to negative psychological outcomes in childhood (Glasheen et al. 2010) and adolescence (Glasheen et al. 2013), while the effects of exposure to paternal anxiety on later child outcomes remain to be investigated. Despite this preliminary evidence on a longitudinal link between parents’ depression and anxiety in the postnatal year and later functioning and psychopathology in their children, we know little about specific developmental pathways that contribute to the emergence of psychopathology in the offspring at specific stages of development. For the time being, it is difficult to delineate the separate and joint contributions of the diverse developmental pathways—including exposure to parental negative emotions and other genetic and environmental factors—to maladaptive child outcomes.

Which aspects of infants’ emotional development we should look for early dysfunction? From a developmental psychopathology perspective, failures of adaptation in certain developmental processes precede the emergence of psychopathology. These are probabilistically linked to the development and course of psychopathology (Sroufe 1990). In our current discussion on the early exposure to parental depression and/or anxiety, we focus on two aspects of infants’ emotional development, namely infants’ expressions of emotion during their early interactions with parents, and their attention to facial expressions of emotion. Alterations in emotional expressions in daily interactions and in attention to negative emotion are considered to be important indices of preverbal infants’ socio-emotional functioning, and correspond to the core domains where dysfunction occurs in childhood and adulthood forms of depression and anxiety (Leppänen 2006; Van Bockstaele et al. 2014). Therefore, these aspects may be particularly useful to consider to detect early effects of exposure and risk for psychopathology in preverbal infants.

What are the transitions and phenomena in infants’ socio-emotional development in the first year of life that are important to consider while discussing the effects of exposure to parental emotions? Two milestones in the socio-emotional domain that we address in the current discussion (Striano and Reid 2006) are the onset of dyadic parent–infant, and triadic parent–infant–object face-to-face interactions (D’Entremont et al. 1997; Messinger and Fogel 2007). Mothers’ positive affect and contingent responding to infants’ expressions of emotion in early dyadic face-to-face interactions are essential for the development of infants’ expression and regulation of emotions in the interpersonal domain in the first half year of life (Als et al. 1979; Cohn and Tronick 1987; Tronick 1989). In triadic parent–infant–object interactions that emerge at the second half of the first year, the parent and infant communicate affective states about an external object, person, situation or event in the environment (i.e., joint attention in the second half of the first year; Carpenter et al. 1998). Between 10 and 14 months, infants start to actively use adults’—most frequently parents’—emotional signals to regulate their behavioral reactions to ambiguous/novel aspects of the environment (so-called social referencing [SR]; Feinman 1982; Feinman et al. 1992). Parents’ emotional expressions in triadic parent–infant–object interactions serve as a basis for infants’ expression and regulation of emotional and behavioral reactions to novelty.

An important milestone that emerges in the transition from the first to the second half year of life in the domain of attention is the emergence of negativity bias in infants’ emotion processing (for a review see Vaish et al. 2008). Studies on behavioral and physiological correlates of infants’ attention consistently reveal that infants start to allocate more attention to negative (vs. positive) stimuli between 5 and 7 months of age (e.g., De Haan et al. 2004; Geangu et al. 2011; Hoehl et al. 2008; Kotsoni et al. 2001; Peltola et al. 2013). A bias for negative emotion was also reported in child behavior in SR situations: Infants change their reactions more strongly when the referee expresses negative as compared to positive emotions. Prioritized processing of negative emotion from parents was suggested to be functional at this period because it enhances the survival chances of crawling/walking infants who may come across potential dangers while exploring the more distant environment (Bertenthal and Campos 1990; Boyer and Bergstrom 2011; Campos et al. 2000; Leppänen 2011). However, repeated exposure to depressed and/or anxious parents’ negative emotions may at the same time influence emerging negativity bias in infants’ emotion processing, and behavior (Vaish et al. 2008). Vaish et al. (2008) suggested that infants’ exposure to positive facial expressions earlier in the first year is necessary to skew the distribution of infants’ exposure to faces toward the positive end, to ensure that negative faces are perceived as more novel and more salient in the second half of the first year. Thus, changes in exposure to parents’ positive and negative affect in the first year may affect the distribution of infants’ overall exposure to positive and negative emotions, and later negativity bias.

In the current review, we incorporate child temperamental dispositions in the discussion of early exposure effects. Child temperamental predispositions are a biologically determined source of variation in infants’ emotional reactivity, expressions and arousal. Infants with a negative temperamental predisposition (also referred as negative reactivity in infancy or behavioral inhibition [BI] from toddlerhood onwards) are more likely to react to change or novelty in the environment with fearful, withdrawn, shy and avoidant responses than infants without such a predisposition (Fox et al. 2005; Kagan and Snidman 1999; Rothbart 2007). Children of depressed and anxious parents are more likely to be behaviorally inhibited than children of reference parents, and behaviorally inhibited children are more likely to develop depression and anxiety (Biederman et al. 1995; Bruder-Costello et al. 2007; Rosenbaum et al. 1993). Infants’ temperamental characteristics may additionally modulate the impact of early environmental adversity on current and later outcomes. Diathesis-stress (Zuckerman 1999) and vulnerability-stress (Ingram and Luxton 2005; Nigg 2006) models predict that infants with negative temperamental dispositions are more vulnerable to the effects of exposure to depression and anxiety from parents. Differential susceptibility to environmental influences hypothesis additionally stresses the buffering role of an adaptive rearing environment for infants with temperamental dispositions (Belsky et al. 2007; Belsky and Pluess 2009).

To address the continuity in exposure effects across clinical and non-clinical parent populations, the current discussion incorporates evidence on exposure to negative emotions in healthy parents without diagnosis in addition to parents diagnosed with depression and/or anxiety. In line with the multi-component model of exposure effects by Goodman and Gotlib (1999), we review evidence on the depression and anxiety-related alterations in parents’ expressions of negative emotion in early parent–infant interactions, together with links of these alterations to infants’ emotional expressions in everyday interactions with parents and to infants’ attention to others’ emotional expressions.^1^ First, we focus on the direct links between infants’ exposure to parents’ negative emotions, and infants’ facial expressions of emotion during daily dyadic face-to-face interactions with their parents. Second, we focus on triadic parent–infant–object interactions to look at the direct links between exposure to parental negative reactions and infants’ emotional and behavioral reactions to novel stimuli. Third, we address indirect effects of exposure by reviewing evidence on the associations of exposure to parents’ emotional facial expressions with infants’ attention to others’ emotional facial expressions. The review finishes with a discussion on potential links between exposure to parental depression and/or anxiety in the first postnatal year and later psychopathology in the offspring, followed by a discussion of mechanisms, future directions and clinical implications.

## Exposure to Parents’ Facial Expressions, and Infants’ Emotional Expressions in Dyadic Parent–Infant Interactions

### Typically Developing Infants

Parents’ facial expressions are predominantly positive, and rarely negative during face-to-face interactions with their infant (between 1 and 9-months of age, e.g., Belsky et al. 1984; Forbes et al. 2004; Kaye and Fogel 1980; Malatesta and Haviland 1982). Mothers are more positive than fathers in these interactions, and infants are more positive in their interactions with their mother than with their father (Belsky et al. 1984; Forbes et al. 2004). Thus, infants’ expressions of positive affect in dyadic face-to-face interactions seem to get tuned into the moment-to-moment differences in mothers’ and fathers’ interactive styles. In experimental manipulations of naturalistic face-to-face situations, parents are asked to switch to a depressive interactive style by maintaining a neutral facial expression for a few minutes before switching back to regular interactions, also referred to as still-face (Tronick et al. 1978). Findings from still-face interactions reveal that 4-month-old infants experience stress, and decrease positive affect in response to decreases in mothers’ and fathers’ positive affect expressions (Braungart-Rieker et al. 1998).

### Infants of Depressed and Anxious Parents

The evidence on depressed parents’ expressions of emotions in dyadic face-to-face situations shows that mothers’ and fathers’ depression may interfere with parents’ positive affect to their infant during early interactions. Parents with depression express less positive, and more negative/flat facial expressions in dyadic face-to-face interactions (Aktar et al. 2016; Beck 1995; Murray et al. 2010; Stanley et al. 2004). Like their depressed parent, 2-to-6-month-old infants of parents with depression express less positive and more negative/flat affect while interacting with their depressed (vs. non-depressed) parent (e.g., Aktar et al. 2016; Campbell et al. 1995; Cohn et al. 1990; Cohn and Tronick 1987; Field 1984). Thus, just like typically developing infants of healthy mothers, infants of parents with depression tune into their parents’ interactive style.

Taken together, the evidence from dyadic interactions of depressed parents and their infants provide support for the first four components of the model by Goodman and Gotlib (1999). Findings pointing at differences between depressed and non-depressed parents’ expressions of emotions in face-to-face interactions are in line with the first and the second components, as they show that depressed parents express less positive and more flat/negative emotions in their interactions and that they may not be able to provide the most optimal interpersonal environment for the offspring. The evidence on the differences in infants’ expressions of emotions with depressed (vs. non-depressed) parents supports the third and fourth components of the model by showing that infants’ socio-emotional development may be affected at least on the level of emotional expressions: Infants of parents with clinical depression seem to copy their depressed parent’s dysphoric interaction style, and become less positive, and more flat/negative in dyadic interactions with the depressed parents (Beck 1995; Campbell et al. 1995; Cohn et al. 1990; Murray et al. 2010; Stanley et al. 2004).

To what extent do infants generalize the depressed interactive patterns that they acquired from depressed parents to the other parent or other adults? Two studies comparing positive affect during infants’ interactions with their depressed (vs. non-depressed) mothers and non-depressed fathers revealed that infants are more positive while interacting with their non-depressed fathers than with their depressed mothers. Edhborg et al. (2003) compared 15-to-18-month-old infants’ interactions with their depressed mothers (measured 2-months postpartum) and non-depressed fathers. More positive interactions were observed in non-depressed father–infant dyads when mothers had postpartum depression. Likewise, Hossain et al. (1994) showed that 3-to-6-month-old infants of depressed mothers interact better with their non-depressed fathers. Non-depressed fathers also received better interaction ratings than depressed mothers. In contrast, a third study by Chabrol et al. (1996) observing 3.5-to-6-month-old infants’ interactions with their moderately depressed (vs. non-depressed) mothers and non-depressed fathers at 2-months postpartum did show similar levels of positive affect in depressed mother–infant and non-depressed father–infant interactions. The inconsistencies in findings may be related to the differences in the severity, and in the time of measuring maternal depression across the studies. Thus, further research is needed to better understand when infants’ depressed interactive styles extend to interactions. A study by Goodman (2008) showed that mothers’ depression in the postpartum period was related to fathers’ depression, and less optimal father–infant interactions at 2-to-3 months’ of age. Thus, it seems that mothers’ depression may in some cases adversely affect fathers’ psychological functioning and interactions with their infant.

One study investigating how 4-month-old infants of depressed mothers interact with other people reported an increase in positive affect expressions of infants during interactions with their nursery teachers, who were more positive than depressed mothers during the interaction (Peláez-Nogueras et al. 1994). In turn, another study by Field et al. (1988) investigating 3-to-6-month-old infants of depressed versus non-depressed mothers in interaction with their mothers versus non-depressed female strangers revealed no differences in infants’ positive affect, or activity level during their interactions with the strangers (vs. mothers). Note that no significant differences were found also between the positive affect of depressed mothers and non-depressed strangers in this study.

Taken together, it seems that infants show more positive affect in dyadic interactions with other familiar partners such as the other parent or teachers. Note that this occurs in the context of the other parent and teachers expressing more positive affect than the depressed mother. In this sense, fathers and other familiar figures may compensate for the depression of the mother by giving the child the opportunity to interact in a more positive manner on a regular basis. However, the results also reveal that fathers who have a partner with depression are themselves more likely to be depressed. Thus, maternal depression may interfere with fathers’ ability to compensate for mothers’ depression in face-to-face interactions via its positive association with paternal depression. As a result, depressed interaction styles in mother–infant interactions may generalize to father–infant interactions.

Studies testing infants’ reactions to depressed parents’ still-face reveal significant alterations in infants’ reactions to still-face, while the findings concerning exact direction of the effect are mixed. Like the effects reported in typically developing samples, Forbes et al. (2004) reported more negative and less positive affect in 3-to-6-month-old infants of parents with (vs. without) depression during both mothers’ and fathers’ still-face. In contrast, other studies found that infants of mothers with depression respond to the mothers’ still-face with more positive, less negative affect and less stress compared to infants of mothers without depression at 3, 4 and 6 months of age (Field 1984; Field et al. 2007; Peláez-Nogueras et al. 1996). Field (1984) explains the results based on familiarity: Infants of depressed parents may be relatively more familiar to parents’ still-face and therefore react with less negative affect to parents’ still-face in the lab.

Compared to parental depression, we know less about how parental anxiety disorder affects parents’ and infants’ emotional expressions in early dyadic face-to-face interactions. The available evidence mostly comes from parents with comorbid depression and anxiety. For example, depressed mothers with high (vs. low) trait anxiety were found to be less positive, and infants of depressed (vs. non-depressed) mothers with high trait anxiety were less positive and more negative at 3 months of age (Field et al. 2005). Thus, high trait anxiety may be linked to a further decrease in depressed mothers’ and infants’ expressions of positive affect. One study reported a decrease in mothers’ facial, vocal and bodily expressions of positive affect when they have high vs. low trait anxiety (after controlling for depression) in face-to-face play interactions with their 10 and 14-month-olds (Nicol-Harper et al. 2007). In contrast, another study revealed that mothers’ and fathers’ expressions of positive and negative affect at 4 months do not change as a function of lifetime parental anxiety symptoms in face-to-face interactions, after controlling for depressive symptoms (Aktar et al. 2016). In turn, higher levels of parental anxiety were related to more positive and more negative affect from infants in this study, suggesting a generally heightened emotional reactivity. But this effect was not directly accounted for by anxious parents’ expressions in the interaction. Anxious parents’ emotional expressions did in fact not differ from those of parents without a diagnosis. Moreover, more anxiety symptoms in parents were related to longer looking, thus to more interest to the mother during the interaction. This study also incorporated infant temperament in the study of face-to-face interaction and revealed no significant link between infants’ observed temperament and their emotional expressions in face-to-face interactions.

Due to comorbidity of anxiety disorder and depression in studied samples, the available evidence revealing more severe alterations in affect expressions of depressed mother–infant dyads cannot disentangle the distinct influences of parents’ depression and anxiety disorder on emotional expressions. Furthermore, because comorbid depression and anxiety diagnoses go together with an overall greater symptom severity (Fava et al. 2004; Lamers et al. 2011), it is unclear whether a more pronounced decrease in mothers’ positive affect is specifically related to high trait anxiety, or to overall greater depression symptom severity. In a sample of anxious mothers with low rates of depression diagnoses, Murray et al. (2007) observed mothers with social anxiety disorder (SAD) and generalized anxiety disorder (GAD) and their infants in face-to-face interactions. Mothers with SAD were less positively engaged, and more anxious during their interactions, while the effect of maternal GAD was not significant. Infants of mothers with GAD and SAD did not differ from infants of mothers without anxiety disorder.

Few observational studies investigated the effects of maternal anxiety without comorbid depression on infants’ and parents’ facial expressions of emotion during dyadic face-to-face interactions. Weinberg et al. (2008) investigated the effects of maternal panic disorder (without depression), and maternal depression on 3-month-old infants’ emotional expressions in a still-face interaction preceded and followed by regular face-to-face interactions. No significant effects of parental panic disorder or depression were found on parents’ and infants’ expressions of affect. Kaitz et al. (2010) investigated the effects of maternal anxiety disorder without comorbid depression on 6-month-old infants’ facial expressions during dyadic interactions (free play, teaching and caregiving episodes). Infants of anxious mothers were not less positive than their peers during the regular face-to-face interactions. Moreover, the duration of matched positive affect in the dyads did not differ between healthy and anxious mother–infant dyads. On the other hand, the findings revealed an overall increase in intensity and frequency of gaze, positive affect, and verbalizations of anxious (vs. reference) mothers, so-called exaggerated behavior. This study also included a still-face interaction. Similar to the earlier results in infants of depressed mothers (Field 1984; Peláez-Nogueras et al. 1996), infants of anxious parents less often reacted with negative affect to mothers’ the still-face in this study.

Reviewed evidence suggests that the alterations in parents’ positive affect expressions in early dyadic interactions are more pronounced in the case of comorbid depression and anxiety disorders (Field et al. 2005) as compared to depression alone. However, the findings from mothers with anxiety disorders without comorbid depression suggest that anxiety disorders alone do not alter parents’ and infants’ expressions of positive affect in everyday face-to-face interactions, even in the case of generalized anxiety disorder (Kaitz et al. 2010; Murray et al. 2007; Weinberg et al. 2008). These findings do not seem to support Goodman and Gotlib’s (1999) components in dyadic interactions. To conclude, the increase in the expressions of negative emotions in anxious parents may not be visible in everyday dyadic parent–infant interactions and may be rather specific to certain anxiety-provoking objects/events/persons in triadic parent–infant–object interactions (like being videotaped in interaction with a stranger in the case of maternal SAD, Murray et al. 2007, 2008).

### Section Summary and Conclusions

Evidence summarized above reveals a direct link between infants’ and parents’ expressions of emotion, stressing the crucial role that exposure to parental positive emotions plays in the regulation of infants’ expressions and communication of affect in their face-to-face interactions in typical development (e.g., Cohn and Tronick 1987; Forbes et al. 2004; Malatesta and Haviland 1982). Surprisingly, studies focusing on the associations between infants’ and parents’ emotional expressions in early dyadic interactions in community samples did not consider the role of infants’ temperamental predispositions. The only study that incorporated infant temperament reported no significant association, revealing that infants’ temperamental dispositions may not always be visible in their observed affect expressions in early dyadic interactions (Aktar et al. 2016). Parallel associations between infants’ and mothers’, and infants’ and fathers’ expressions of positive affect in typical development reveal that infants adapt their affect expressions to the differences in mothers’ versus fathers’ moment-to-moment expressions of positive affect in dyadic face-to-face interactions (Forbes et al. 2004). In line with the findings suggesting a direct influence of parents’ positive affect expressions in this period on infants’ expressions of positive affect in typical development (Braungart-Rieker et al. 1998; Forbes et al. 2004), infants are less positive, and more flat and more negative in dyadic interactions with the depressed parents (Beck 1995; Campbell et al. 1995; Cohn et al. 1990; Murray et al. 2010; Stanley et al. 2004). Available evidence comparing mother–infant and father–infant face-to-face interactions in families where the mother is depressed reveals that infants are more positive in their interactions with their non-depressed fathers. Infants are also more positive with familiar figures who express more positive affect than the depressed mother. The other parent, and other familiar figures may thus compensate for parents’ depression in face-to-face interactions (Edhborg et al. 2003; Hossain et al. 1994; Peláez-Nogueras et al. 1994) and thus provide the infant with a more positive early interactive environment. However, findings also reveal that due to its positive association with paternal depression, maternal depression may in some cases interfere with father–infant interactions and fathers’ ability to compensate for mothers’ depression in face-to-face interactions (Chabrol et al. 1996; Goodman 2008).

Preliminary evidence from the observational studies comparing parental anxiety disorders without comorbid depression versus no parental diagnosis revealed no significant differences in parents’ and infants’ expressions of affect in naturalistic face-to-face interactions. In turn, infants of anxious mothers seem to react less negatively when mothers stop responding in still-face interactions.

## Exposure to Parents’ Facial Expressions and Infants’ Emotional Expressions and Reactions to Novelty in Triadic Parent–Infant–Object Interactions

### Typically Developing Infants

The effects of mothers’ positive and negative emotional expressions on infants’ behavioral and emotional reactivity to novel stimuli have been extensively studied in social referencing (SR) situations at the end of first year of life (see Feinman et al. 1992 for a review). Findings from these observational studies provide support for a direct causal influence of parents’ expressions of negative emotions at the end of the first year on infants’ affect and behavior: Infants interact with novel stimuli less and manifest more negative affect (i.e., fear) and more avoidance when the referee expresses negative (vs. positive) emotions about these stimuli.

Two studies investigated the effect of parents’ anxious signals in SR situations in typically developing infants to understand early mechanisms of infants’ fear learning from anxious parents in infancy. De Rosnay et al. (2006) investigated the effects of mothers’ expressions of anxiety toward a stranger on 12-to-14-month-old infants’ stranger fear and avoidance. Parents without anxiety disorders were trained to behave in socially anxious ways in a stranger SR paradigm. In this paradigm, a stranger first engages the parent in a conversation while the infant is watching the interaction. At the end of the parent–stranger interaction, the stranger makes a gradual approach toward the infant and picks him/her up. Findings from this study revealed that expressions of maternal anxiety toward the stranger can trigger infants’ avoidance of the stranger. This effect was moderated by infants’ fearful temperament, such that the link between maternal negative reactions and infants’ avoidance of the stranger was stronger for infants with high levels of fearful temperament (De Rosnay et al. 2006). The second observational study investigated the links of mothers’ and fathers’ expressions of anxiety to 10-to-15-month-old infants’ fear and avoidance in the visual cliff (Möller et al. 2014). In the visual cliff paradigm, infants, who are placed on the shallow end of the cliff, are encouraged to crawl toward their parent who stands at the deep end (Sorce et al. 1985). Möller et al. (2014) found that fathers’, but not mothers’ anxious signals predict temperamentally fearful infants’ avoidance of the visual cliff. Taken together, findings from both studies reveal an interplay between parents’ expressions of anxiety and infants’ fearful temperament in SR situations. In line with diathesis-stress (Zuckerman 1999) and vulnerability-stress (Ingram and Luxton 2005; Nigg 2006) models, the findings show that temperamentally fearful infants are more vulnerable to the expressions of parents’ anxiety in SR contexts.

Regarding effects of mothers’ versus fathers’ emotional signals in SR situations, an earlier study that simultaneously tested mothers and fathers as referees did not find a significant difference on 12-month-old infants’ reactions to novel toys with mothers and fathers (Hirshberg and Svejda 1990), while the findings of Möller et al. (2014) suggest that fathers’ but not mothers’ expressions of anxiety predict avoidance of the cliff. This discrepancy in the findings may possibly be related to the differences in the age of the samples, or in the testing (mothers and fathers being tested together vs. separately), or in the type of threat (i.e., falling vs. being harmed by an ambiguous object). Nevertheless, findings from both studies suggest a direct link between parents’ emotional expressions in SR situations and infants’ reactions toward novel/ambiguous aspects of the environment.

### Infants of Depressed and Anxious Parents

SR studies in clinical samples have predominantly focused on the effects of anxious parents’ expressions of fear and anxiety, rather than the effect of depressed parents’ dysphoric style on infants’ reactions to novelty during triadic parent–infant–object interactions. Although the associations between parents’ emotional signals and infants’ reactions to novelty have not been investigated in the context of SR, an earlier study investigating infants’ object exploration and emotional expressions reported that 11-to-14-month-old daughters (but not sons) of depressed (vs. non-depressed) parents express less positive and more negative affect than infants of non-depressed mothers in triadic parent–infant–object interactions (Hart et al. 1998). Moreover, infants of depressed mothers were less likely to explore the toy objects. Thus, it seems that maternal depression may affect parents’ and infants’ facial expressions similarly in dyadic parent–infant and triadic parent–infant–object interactions. Furthermore, exposure to parental depression seems to be linked to a decrease in infants’ exploring of the novel stimuli in the environment. In a recent discussion, Peláez et al. (2013) suggested that flat affect in depressed parents limits their ability to provide threat/safety signals to their infants in SR situations. Gewirtz and Peláez-Nogueras (1992) further suggest that as a result of parents’ limited availability in SR situations, infants will be less likely to use mother as a source of information in SR situations. This idea awaits further investigation of parents’ and infants’ expressions of emotion in SR situations in infants of depressed parents.

The first studies focusing at the effect of anxiety disorder on parents’ and infants’ expressions of negative emotion in SR situations investigated the links between socially anxious parents’ expressions of anxiety and infants’ fear and avoidance of strangers to shed light on the early intergenerational transmission of social anxiety (Aktar et al. 2013; Murray et al. 2008). Murray and colleagues investigated SR processes in a longitudinal design with socially anxious (vs. reference) mothers and their infants at 10 and 14 months in the stranger SR paradigm. Socially anxious mothers expressed more anxiety than reference mothers both at 10 and 14 months. Furthermore, highly behaviorally inhibited infants of mothers with SAD showed a longitudinal increase in avoidance of strangers in SR situations from 10 to 14 months. In a later replication and extension of this study to non-social SR situations and to parents with non-social types of anxiety disorders, Aktar et al. (2013) found that parents’ expressions of anxiety in the SR situations predict 12-month-old infants’ avoidance in SR situations (in interaction with infants’ behavioral inhibition), rather than parental lifetime (social or non-social) anxiety diagnoses. Thus, 12-month-old infants’ avoidance of novelty was directly related to their environmental exposure to anxious responses in the SR situations, rather than the presence/absence of an anxiety diagnosis. Consistent with previous evidence (De Rosnay et al. 2006; Murray et al. 2008), infants with moderate-to-high levels of fearful temperament (observed behavioral inhibition) were more avoidant of novel stimuli when parents expressed more anxiety in SR situations. In a follow-up study of this sample at 30-months, a different pattern emerged: Parental lifetime SAD rather than parents’ expressions of anxiety predicted toddler’s avoidance. Thus, the direct link between parents’ expressions of anxiety and children’s avoidance may be specific to the end of first year of life in SR situations (Aktar et al. 2014). Moreover, children of parents who had lifetime comorbid social and other anxiety diagnoses, and who expressed more anxiety at 12 months were more avoidant of novel stimuli at 30 months. Thus, comorbid social and other anxiety diagnoses in parents may create a vulnerability for prolonged effects of earlier exposure to parental anxiety in SR situations in toddlerhood. At 12 and 30 months, no significant differences were found in this sample in the associations between mothers’ and fathers’ expressions of anxiety. Thus, fathers’ emotional expressions seem to be as important as mothers’ for infant’s fear learning. Taken together, the available evidence from clinically anxious parent samples is in line with Murray et al. (2009) and with the first four components of the Goodman and Gotlib’s (1999) model. Parents with SAD express more anxiety in triadic interactions with novelty, and this seems to make them less than optimal referees in the SR situations (first and second components) as infants of anxious parents are likely to be repeatedly exposed to high levels of parental anxiety expressions during confrontations with ambiguous stimuli. Findings also suggest that witnessing parents’ anxious behavior in SR situations at the end of first year may concurrently and prospectively increase infants’ avoidance of novelty (third and fourth components) and may thereby contribute to parent-to-infant transmission of anxiety (Aktar et al. 2013; Murray et al. 2008).

### Section Summary and Conclusions

The evidence reviewed above shows that infants’ exposure to parents’ expressions of emotion toward novel objects/people/events in the environment has a direct effect on infants’ avoidance of these novel stimuli/situations at the end of the first year, both in community and in clinical samples (Aktar et al. 2013; De Rosnay et al. 2006; Möller et al. 2014; Murray et al. 2008).

Findings from experimental and semi-experimental SR studies in community samples and in clinical samples consistently suggest that infants’ own temperamental dispositions (i.e., behavioral inhibition) moderate the effects of exposure to parents’ expressions of anxiety on infants’ emotional and behavioral responses to ambiguity in SR situations (Aktar et al. 2013; De Rosnay et al. 2006; Möller et al. 2014; Murray et al. 2008). Consistent with the predictions of diathesis-stress, vulnerability-stress and differential susceptibility models (Belsky and Pluess 2009; Ingram and Luxton 2005; Nigg 2006), temperamentally inhibited/difficult/fearful infants are more sensitive to the effects of exposure to parents’ negative emotions in triadic contexts at the end of first year. Moreover, exposure to parents’ expressions of anxiety from parents with comorbid social and other anxiety diagnoses in infancy predicts more avoidant reactions to novelty in the offspring 1.5 year later (Aktar et al. 2014). Thus, exposure to anxiety expressions of parents with more severe forms of anxiety disorders at the end of first year may have prolonged effects in the offsprings’ avoidance of novelty in toddlerhood.

## Exposure to Parents’ Facial Expressions and Infants’ Attention to Others’ Emotional Expressions in Dyadic and Triadic Contexts

### Typically Developing Infants

In an ERP study, De Haan et al. (2004) investigated how mothers’ reports of their positive and negative affect, and of their infants’ positive and fearful temperament relate to 7-month-old infants’ attention to happy and fearful faces. They found that infants’ negativity bias was moderated by mothers’ positive (but not negative) affect: Infants of mothers who were high in positive affect looked longer to fearful faces (thus showed a negativity bias), while infants of mothers with low positive affect did not show any looking preferences. Thus, it seems that more exposure to mothers’ happy facial expressions was linked to less interest to happy faces. ERP correlates of infants’ attention in this study revealed that temperamentally fearful infants devoted more attention (a larger Nc component, an ERP index of attention allocation) to fearful than to happy facial expressions. Moreover, temperamentally positive infants of highly positive mothers allocated more attention (a larger Nc component) to fearful than happy facial expressions. Thus, it seems that temperamentally positive infants react less strongly to happy faces in ERP indices of attention when they have been more frequently exposed to positive emotions from their mothers. Taken together, findings from this first study support the idea that the variation in infants’ exposure to parents’ positive affect, together with infants’ temperament, may be linked to infants’ attention allocation to emotional stimuli in typical development.

Jones et al. (2013) investigated the links between 3.5-month-old infants’ attention (or interest) to mothers’ neutral faces, and parents’ depression, anxiety and stress levels. They found that more anxiety (but not depression or stress) is linked to less interest to mothers’ (but not to strangers’) neutral faces. Thus, previous findings revealing a lower likelihood of negative reactions to mothers’ still-face in infants of anxious parents (Kaitz et al. 2010) may be related to a decrease in attention to mothers’ neutral expressions. The findings suggest that heightened exposure to parents’ negative emotional expressions in the case of parental anxiety may be related to less attention to mothers’ neutral faces.

### Infants of Depressed and Anxious Parents

Studies in infants of clinically depressed mothers reveal significant links of exposure to mothers’ depressive moods and infants’ attention to facial expression of emotion. Infants of depressed parents show differences in behavioral correlates of attention to sad and happy faces that are indicative of an increased familiarity to sad faces (see Field et al. 2009 for a review). At three and six months of age, infants of depressed mothers spend less time looking at sad faces as compared to infants of non-depressed mothers (Field et al. 1998). Another study reported less interest to both happy and sad faces in infants of depressed mothers, independent of who (mother vs. stranger) poses the expressions (at 5 months of age, Diego et al. 2004). Infants of depressed parents were additionally shown to be more likely to attend to mothers’ happy expressions in face-to-face interactions at 6 months (Striano et al. 2002), and to habituate more slowly to happy facial expressions as compared to infants of non-depressed mothers at 3 months (Hernandez-Reif et al. 2006). Moreover, unlike typically developing infants, 3-to-6-month-old infants of depressed mothers fail to discriminate happy from neutral faces following habituation (Bornstein et al. 2011). Taken together, the evidence from infants of depressed parents in dyadic contexts reveals that more exposure to mothers’ sad faces is indirectly linked to less attention to sad faces in infants. This indirect association is in line with the fourth component of the model by Goodman and Gotlib (1999) that states that changes in exposure will be affecting children’s development. However, it also shows that these changes on the domain of attention may not be per se adverse.

The first evidence on the effect of parental anxiety disorders on infants’ attention has come from Creswell et al. (2008, 2011) who investigated how parental SAD relates to infants’ attention to low versus high-intensity negative facial expressions in a clinical sample. They compared the differences in initial orientation, and total looking time to fearful and angry facial expressions in infants of mothers with (vs. without) SAD. Infants of socially anxious mothers were more likely to orient, and to look at low (vs. high) intensity fearful faces at 10-weeks of age, whereas infants of reference mothers showed the opposite pattern, that is, a bias for high-intensity fearful faces. There were no group differences in infants’ interest to anger: all infants independent of parents’ social anxiety showed more looking to high (vs. low) intensity angry faces. Interestingly, infants’ observed temperament and of parents’ observed expressions of anxiety with a stranger did not account for differences in infants’ visual interest to fearful faces. In a follow-up of this sample, an early preference for high-intensity fear faces (at 10 weeks of age) predicted more anxiety symptoms in the offspring of mothers with social anxiety disorders, while a preference for low-intensity fear faces predicted less child anxiety at 2 years (Creswell et al. 2011). Infants of index mothers showed the opposite pattern with a preference for high-intensity fearful faces being linked to less anxiety at 2 years. This first evidence on direct links between early attention and later anxiety outcomes in the offspring of socially anxious mothers suggests that the links of offspring’s attention to facial expressions to later anxiety may differ as a function of maternal SAD diagnosis. In other words, adaptive and maladaptive attention trajectories in infants’ emotion processing seem to be different in the presence of an anxious mother. Avoidance of high-intensity fearful faces seems to be the general and adaptive response to having an anxious mother. Thus, a preference for high-intensity fearful faces is adaptive in typical development, but maladaptive in children of socially anxious mothers.

### Section Summary and Conclusions

Evidence reviewed above supports the idea that infants’ exposure to positive emotions from parents in this period is linked to infants’ attention to strangers’ positive emotions in typical development (De Haan et al. 2004). Increases in temperamentally positive infants’ exposure to mothers’ positive mood seem to be linked to a decrease in infants’ attention to happy (vs. fearful) facial expressions, along with an increase in attention to strangers’ fearful expressions at 7 months. Moreover, infants’ exposure to mothers’ sad emotions in the case of clinical maternal depression seems to be linked to less attention to sad (vs. happy) facial expressions, and more attention to happy facial expressions (Field et al. 1998; Striano et al. 2002). In other words, in contrast to normally developing infants who are most familiar with happy facial expressions, infants of clinically depressed parents seem to be more familiar with sad faces, and to perceive happy faces as more novel.

Higher levels of maternal anxiety (but not depression or stress) was found to be related to less interest to mothers’ (but not to strangers’) neutral faces in a community sample, while how exposure to parental anxiety may alter infants’ attention to mothers’ and others’ negative facial expressions remains to be further investigated (Jones et al. 2013). The only longitudinal evidence from clinically anxious mothers reveals that the links of offspring’s early attention to later anxiety may be moderated by the presence of SAD in mothers. Taken together, the evidence support the fourth component of the model by Goodman and Gotlib (1999) while revealing that depression and/or anxiety-related alterations in infants’ exposure do not necessarily have adverse consequences for later functioning in all children.

To sum up, the studies reveal that more exposure to a given emotion from parents is linked to less attention to that emotion among infants of parents with and without depression/anxiety diagnoses. Findings from infants of mothers with SAD reveal one adaptive and one maladaptive attention trajectory: Less interest to high-intensity fearful faces from infants is adaptive in the case of parental anxiety disorders, while more interest to high-intensity fearful faces is maladaptive. Thus, only a subgroup of children with an attentional bias for strong negative expressions from the mother are at increased risk for anxiety.

## Discussion

The present study brought the first postnatal year into the spotlight to present an overview of little research evidence available on the links of infants’ exposure to parents’ negative emotions to infants’ emotional development, and to guide future research on parent-to-child transmission of depression and anxiety in this early, developmentally sensitive period. We focused on infants’ everyday interactions with the parents, and their attention to facial expressions as indices of child emotional development (see Table 1 for an overview of key findings). Our review shows parallels between parents’, and infants’ expressions of negative facial expressions in early interactions. That is, infants are less positive and more negative/flat when exposed to less positive and more negative/flat affect from depressed parents in face-to-face interactions (e.g.: Campbell et al. 1995; Cohn et al. 1990; Forbes et al. 2004). Similarly, when exposed to parents’ anxious reactions to novel stimuli in SR situations, infants become more avoidant of these stimuli if they are temperamentally fearful (Aktar et al. 2013; De Rosnay et al. 2006; Möller et al. 2014; Murray et al. 2008). Thus, infants’ fine-tuning into the parents’ expressions of emotion is essential for infants’ survival, development and socialization in the first year of life, whereas it may create a vulnerability for the effects of exposure to negative emotions in infants who have early temperamental dispositions, and/or parents with clinical depression and/or anxiety. It is important to note here that the limited number of studies that incorporated child temperamental dispositions provided support for diathesis-stress and vulnerability models as they revealed that temperamental anxiety dispositions enhance the influence of parental negative signals on child reactions (De Rosnay et al. 2006; Murray et al. 2008).

**Table 1:** An overview of the preliminary findings from the current review

*Preliminary findings*
*Dyadic parent–infant interactions*
Depression in mothers and fathers is linked to less positive and more negative/flat affect in parents and in infants
Children of depressed mothers interact more positively with their non-depressed fathers or other familiar figures than they do with depressed mothers
Comorbid anxiety in depressed parents is linked to a more pronounced decrease in positive affect in parents and children
Mothers and fathers with anxiety disorders (without comorbid depression) do not differ from parents without diagnosis in their expressions of positive and negative affect
*Triadic parent–infant–object interactions*
Daughters, but not sons, of depressed mothers are more positive and less negative than infants of non-depressed mothers
Infants of depressed mothers are less likely to engage in toy exploration
Social anxiety disorder in mothers and fathers is related to more expressed anxiety in parents during SR
More expressed anxiety in fathers and mothers is related to more avoidance of novelty in children with temperamental dispositions for anxiety
*Attention to emotion*
More exposure to sad faces from depressed mothers is indirectly linked to less attention to sad faces in infants
More exposure to fearful faces from anxious parents is indirectly linked to less interest to high-intensity fear faces in infants

Our review reveals distinct associations of parents’ depression versus anxiety diagnoses with parents’ and infants’ expressions of negative emotion in their interactions. Infants of depressed parents are exposed to less positive and more negative/flat affect in early interactions (Aktar et al. 2016; Campbell et al. 1995; Cohn et al. 1990). In contrast, expressions of affect in parents with anxiety disorders (without comorbid depression), and in their infants, do not seem to differ from reference parents in dyadic interactions (Aktar et al. 2016; Kaitz et al. 2010; Murray et al. 2007; Weinberg et al. 2008). Thus, the idea that mothers with anxiety disorders would expose their infants to more negative emotions was not supported in dyadic interactions. The alterations in anxious parents’ emotional expressions seem to be only visible in triadic interactions involving the specific situations that trigger anxiety on the side of the parent (Aktar et al. 2013; Murray et al. 2008).

The evidence on the links between infants’ exposure to parents’ facial expressions of emotions and their attention to emotional facial expressions showed that infants’ exposure to mothers’ positive affect is related to their attention to strangers’ positive versus negative emotions in typical development (De Haan et al. 2004). The link between more exposure to a certain emotion from the mother and less attention to others’ expressions of that emotion seems to hold across infants of mothers from community and clinical samples of parents. That is, typically developing infants who have been exposed to high levels of maternal positive affect in everyday life seem to attend less to the happy (vs. fearful) facial expressions (De Haan et al. 2004), while infants of depressed mothers who are exposed to increased levels of flat/negative faces of their mother seem to attend less to sad (vs. happy) facial expressions (Field et al. 1998, 2009; Hernandez-Reif et al. 2006; Striano et al. 2002). This is likely to be due to relatively increased familiarity/decreased novelty of sad faces for infants of depressed parents. Although it was tested with neutral expressions, the study by Jones et al. (2013) also reveals a link between more anxiety in parents and less attention to mothers’ facial expressions. Note that this pattern is the opposite of an anxious/depressed information processing style defined by more attention to negative stimuli in parents and children with depression and anxiety (Leppänen 2006; Van Bockstaele et al. 2004). As preliminary evidence by Creswell et al. reveals in the case of parental SAD (2008, 2011), less attention to negative expressions may be an adaptive response that shields the infants from repeated exposure to parents’ negative moods in early interactions. In contrast, a subgroup of children with attention biases toward negative stimuli seem to be at risk for later anxiety disorders.

Reviewed evidence on the exposure effects in the first year of life is overall in line with the multi-component model by Goodman and Gotlib (1999). Namely, it shows that parents with depression and anxiety express more negative/flat emotional expressions in everyday interactions with their infants. Due to experience-dependent nature of early emotional development, and parents’ heightened negative affect expressions, depression and anxiety diagnoses may make parents less than optimal social partners in early interactions. The interpersonal environment that they provide may be less than optimal for children’s emotional and social development. The evidence revealing more negative and flat affect and less attention to others’ negative emotion in infants of depressed and anxious parents reveals that this suboptimal interpersonal environment may affect infants’ emotional and social development. The direct links between parents’ and infants’ emotional expressions in everyday interactions are in line with the idea that children acquire negative and avoidant interactive styles that look like negative behaviors and moods of depressed/anxious parents. In turn, the indirect link between infants’ overall exposure to parental negative emotion and attention to others’ emotional expressions reveal a negative, rather than a positive association. In other words, when exposed to high levels of a certain emotion from the parent, infants seem to attend less to that emotion. We conclude that exposure to parents’ negative emotions does not necessarily have only adverse links to infants’ emotions’ processing, but it may also desensitize infants to others’ negative emotions, thus pushing infants’ processing toward the non-anxious, non-depressed information processing style. The links between infants’ expression and attention to emotion remain to be investigated in future multi-method longitudinal studies combining the observation of everyday interactions with the measurement of infants’ attention to emotional stimuli.

In contrast, the current review does not allow any evidence-based conclusions on the last component of the model by Goodman and Gotlib (1999), i.e., on the links of exposure-related alterations in infants’ emotional development and the development of anxiety disorders and depression in childhood or adolescence. It remains unknown whether the increased negative/flat affect expressions in infants is a predisposition for later development of depression and anxiety. Longitudinal designs incorporating the measurement of socio-emotional development together with the diagnostic measurements that identify depression and anxiety in the offspring are needed to answer this question.

Our conclusions on the links of exposure to parental negative emotions to infants’ emotional development are only preliminary as only few studies addressed exposure effects in infancy, mostly in simple correlational and cross-sectional designs. This precludes strong conclusions on a causal longitudinal association between exposure to parental depression/anxiety in the first year and later psychological outcomes in the offspring. The available longitudinal work is limited to short-term prospective designs that, most of the time, did not directly assess clinical outcomes. Second, because the reviewed evidence is from highly educated, higher-middle SES parents and their infants in Western countries, the current conclusions have limited generalizability. Considering that parents’ emotional expressions in everyday interactions are part of socialization practices largely shaped by culture, it is crucial to consider the variation accounted for by cultural differences in infants’ emotional development (Halberstadt and Lozada 2011). Moreover, recent evidence reveals that infants’ attention to emotions and emotional expressions in everyday interactions differ as a function of SES, illustrating the importance of including SES in future studies as a potential source of individual differences (Clearfield and Jedd 2013; Tacke et al. 2015). In the next section, we put the findings in the context of intergenerational transmission by addressing potential mechanisms that may link exposure effects in infants’ emotional expressions, behavioral reactions and infants’ attention to the intergenerational transmission of depression and anxiety in infancy.

### Intergenerational Transmission of a Depressive Interpersonal Style from Parents in Infancy

The findings consistently show that depressed mothers trigger a depressed interaction style characterized with less positive and more negative/flat affect from their infants in early interactions. Field and colleagues (Field 1984; Field et al. 1998) suggest that this may generalize to infants’ expression of emotion in their interactions with other adults and contribute to an overall less positive and more negative affective tone from the infant. The environmental transmission of this depressive interpersonal style from parents to infants may contribute to the intergenerational transmission of parental depression.

The association between more exposure to parents’ depressed mood and the less attention allocation to happy (vs. sad) expressions from strangers implies a different influence of exposure to parental depression on infants’ attention allocation (Field et al. 1998, 2009; Hernandez-Reif et al. 2006; Striano et al. 2002). That is, infants of depressed parents attend to others’ positive rather than sad facial expressions that they are already familiar with from their interactions with mothers. Infants of depressed parents also show more interest to mothers’ happy facial expressions (Striano et al. 2002). More attention to positive expressions may be adaptive for infants of depressed parents as this would increase infants’ chances for exposure to more positive emotions. In other words, infants’ increased attention to happy expressions in this period may be a protective mechanism that shields the infant from exposure to negative or flat emotional expressions of depressed mothers by directing their attention to mothers’ and others’ positive expressions. In turn, an interest to negative or flat expressions of the depressed mother may be an early risk in the intergenerational transmission of depression.

#### Mechanisms of Transmission

Field (1984) suggested two potential mechanisms for the early transmission of a depressive interpersonal style from depressed parents to children in infancy. First, infants’ may acquire a dysphoric interaction style by mirroring parents’ expressions of emotion during dyadic face-to-face interactions. In line with this idea, positive associations were reported not only between infants’ and mothers’ expressions of specific emotions, but also between infants’ and mothers’ use of eye and brow muscles while expressing these emotions during face-to-face interactions (Malatesta and Haviland 1982). Second, the decreased positivity of depressed parents may render the dyadic interactions less arousing, exposing infants to lower than optimal levels of stimulation (Field 1984). In addition to a decrease in positive arousal specifically stemming from the decreased positivity in parents’ emotional expressions, depressed parents seem to provide overall a less stimulating environment for infants. For example, depressed mothers are less likely to tell stories to their infants, while depressed fathers are less likely to play with, and sing to their infant (Paulson et al. 2006).

### Intergenerational Transmission of Anxious/Avoidant Reactivity Patterns from Parents in Infancy

The findings consistently reveal a significant effect of exposure to parental expressions of anxiety on infants’ emotional expressions/reactions to novel/ambiguous stimuli at the end of the first year (Aktar et al. 2013; De Rosnay et al. 2006; Möller et al. 2014; Murray et al. 2008). The increase in infants’ expressions of fear and behavioral avoidance toward novel/ambiguous stimuli was found to be especially more pronounced for infants with a temperamental disposition for anxiety. Thus, temperamental dispositions seem to modulate the link of exposure to parental anxiety to child avoidant reactions, and exacerbate the effects of exposure to parental anxiety.

#### Mechanisms of Transmission

The mechanisms explaining the links between repeated exposure to fearful/anxious expressions from parents with anxiety disorders and the resulting increase in infants’ fear/avoidance responses in infancy have been operationalized within the framework of fear acquisition models (Fisak and Grills-Taquechel 2007; Murray et al. 2009; Rachman 1977). Together with the learning experiences that involve direct confrontations with threat (i.e., classical conditioning), indirect acquisition of fear via verbal or non-verbal forms of social learning is among the major pathways for fear acquisition (Olsson and Phelps 2007; Rachman 1977). Classical conditioning and observational learning precede language (and instructional learning) in our evolutionary history and seem to rely on the same emotional brain systems (i.e., amygdala-mediated fear learning pathways, Olsson and Phelps 2007) in the brain. In line with this, vicarious learning is often conceptualized as a form of classical conditioning where fearful/anxious signals from parents serve as an unconditional stimulus, triggering fearful/anxious reactions. Following the pairing of these signals with ambiguous stimuli, the ambiguous stimuli become conditioned stimuli, evoking fearful/anxious reactions from the child (see Askew and Field 2008 for a review). Considering that anxious parents, by definition, have more frequent and intense experiences of anxiety triggered by ambiguity, parents with anxiety disorders are more likely to provide threat signals increasing the chances for associative learning of fear, leading to an increase in infants’ negative reactions. In addition to fear responses, infants’ observational learning of anxious parents’ anxious/avoidant behavioral styles via repeated exposure, and the resulting increase in infants’ overall perception of threat in the environment were suggested to contribute to the social transmission of anxiety from parents with anxiety disorders (Fisak and Grills-Taquechel 2007; Rapee 2001). Finally, anxious parents’ reinforcement of infants’ anxious/avoidant behaviors has been stressed as a form of operant conditioning. (Fisak and Grills-Taquechel 2007). Differently from reference parents who reinforce approach and exploration of novelty with their infants, parents with anxiety disorder can reinforce anxious/avoidant coping styles due to their own excessive reactions, or to previous aversive experiences in anxiety-provoking situations.

A preference for low-intensity negative expressions was reported in infants of parents with SAD (Creswell et al. 2008, 2011). Infants’ decreased attention to high-intensity negative faces in the case of maternal anxiety may be part of a protective mechanism that protects the infant from exposure to mothers’ fearful/anxious expressions by directing the attention away from negative emotions. Less exposure to high-intensity negative faces from others may help in bringing the distribution of overall exposure to positive and negative affect in infants of anxious parents closer to those of typically developing infants.

### Exposure to the Other Parents’ Emotional Expression as a Moderator of Exposure to Depression and Anxiety

Exposure to mothers’ and fathers’ emotional expressions mostly happens together in development and jointly determines the distribution of infants’ overall exposure to emotions. This is why it is important to look at the joint effects of infants’ exposure to emotions from mothers and fathers. The implicit assumption that mothers are more important than fathers seems to considerably restrict our knowledge about fathers in this field of research, just like in other domains of developmental psychopathology. Evolutionary models of parenting challenge this assumption by assigning fathers a differential role in typical development, and in the development of psychopathology (Bögels and Perotti 2011; Bögels and Phares 2008). As our review of the evidence reveals, the effects of exposure to mothers’ and fathers’ exposure effects have rarely been investigated together. Considering our limited knowledge on the interplay of gender of the parents with psychopathology, in this section, we simply assume mothers’ and fathers’ influences are equivocal, and we refer to the secondary caregiver parent as ‘the other parent’.

Two important aspects that are likely to determine whether the influence of exposure to the other parents’ emotional expressions is a risk or a buffer are the other parent’s involvement in infant care and the other parent’s psychopathology, especially depression and/or anxiety. Involvement determines the extent of infants’ exposure to the parents’ expressions of emotion, while psychopathology determines the proportion of exposure to positive versus negative emotions from the other parent. Fathers’ availability and mental health appear as a moderator of exposure to mothers’ depression in the developmental model of intergenerational transmission of depression by Goodman and Gotlib (1999). In accordance with this model, we suggest that exposure to the other parents’ emotions can become a risk factor in infancy when higher frequencies of exposure to the other parent co-occur with depression and anxiety in the other parent. Thus, when the other parent is highly involved in the infants’ care and has a depression and/or anxiety, the other parent may exacerbate the effect of depression and anxiety in one parent. Due to assortative mating, parents with depression and anxiety disorders are more likely to choose partners that do have similar types of psychopathology, resulting in significant associations between couples’ depression and anxiety diagnoses (Goodman 2004; Matthey et al. 2003), and in higher risk for psychopathology in the offspring (Merikangas et al. 1988a; b). Psychopathology in the other parent not only contributes to a more pronounced genetic and biological risk for intergenerational anxiety, but also to more pronounced alterations in the overall distribution of infants’ exposure to parental emotions when the other parent is highly involved in care. In line with this idea, a study in a clinical sample of depressed mothers revealed that exposure to postpartum maternal depression is linked to later internalizing and externalizing problems in the offspring in the toddlerhood, only in the presence of paternal psychopathology (Dietz et al. 2009). Furthermore, a community study reported that exposure to fathers’ depression in infancy strengthens the association between early exposure to mothers’ depression in infancy and children’s behavioral problems in the kindergarten only if the father is involved in care (Mezulis et al. 2004). These findings suggest that the other parents’ depression, together with his/her involvement, may create more pronounced alterations in infants’ exposure and lead to poorer outcomes when both parents are depressed. Similarly, when both the mother and the father have anxiety, and are involved in infants’ care, a higher frequency of exposure to parental expressions of anxiety in anxiety-provoking situations would be expected to be associated with less optimal outcomes than in the case of a single parent having anxiety disorder.

Alternatively, in cases where the other parent has no diagnosis of depression or anxiety, and is involved in infant care, exposure to the other parents’ emotional expression may provide the infants the opportunity to interact more positively with the other parent on a regular basis, which may help to bring the distribution of infants’ overall exposure closer to the typically developing infants’ exposure. The findings suggesting that fathers compensate for mothers’ depression by expressing more positive affect than their depressed partners in their face-to-face interactions with the infant support this idea (Edhborg et al. 2003; Hossain et al. 1994). However, contrasting evidence revealing no significant differences in the interactions of depressed mothers and non-depressed fathers with their infants (Chabrol et al. 1996), and less optimal interactions in non-depressed fathers when the mother is depressed (Goodman 2008) show that the other parent may not always be able to compensate for partners’ depression and anxiety. Thus, the other parent’s ability to compensate may depend on several factors including their own depression and the negative influences of living with a depressed partner on fathers’ own functioning and on the marital relationship quality. Future studies focusing on the joint effect of exposure to maternal and paternal emotions should thus consider dynamic transactions between the other parents’ involvement, psychopathology, along with other factors related to the marital functioning.

### Parents’ Emotion Coaching as a Potential Mechanism in the Intergenerational Transmission of Depression and Anxiety

Our review on parent and infant emotional expressions and reactions in everyday interactions illustrates the extent to what infants rely on parents’ skills to regulate their negative emotions in the first year of life. Considering this early regulatory function of parents’ emotional reactions and expressions on infants’ emotion in early interactions (also see Bornstein 2013), parent-to-offspring transmission of emotion regulation difficulties may play a role in parent-to-offspring transmission of depressed or anxious moods (Campbell-Sills and Barlow 2007). Impairments in the down-regulation of negative emotion characterize both childhood and adulthood forms of depression and anxiety disorders (Cisler et al. 2010; Joormann and Gotlib 2010). Because depressed and/or anxious parents have difficulties in down-regulating their own negative emotions, they may also be less likely to detect, accept, or acknowledge infants’ experience of negative emotions in everyday interactions. Emotion coaching is the term that refers to the extent to which parents welcome infants’  negative emotions and provide guidance for infants’ recognizing, understanding and dealing with the experience of negative emotions (Gottman et al. 1997). Higher levels of parental emotion coaching have been linked to better emotion regulation skills, better adjustment and less internalizing problems in childhood and adolescence (Gottman et al. 1996; Stocker et al. 2007). Moreover, in line with differential susceptibility to environmental influences hypothesis, recent evidence reveals that children benefit from parents’ emotion coaching especially if they have a disposition for negative emotion (Dunsmore et al. 2016). This evidence on a buffering/protective function of parental emotion coaching on child functioning in older children of non-diagnosed parents raises the question of whether better emotion coaching in depressed and/or anxious parents may alleviate some of the observed alterations in depressed/anxious parents’ emotional expressions with their infants in daily life. Considering higher rates of emotion regulation difficulties in depression and anxiety, and higher rates of negative temperamental dispositions in children of depressed/anxious parents, it is important to investigate how better parental emotion coaching skills may be related to parents’ affect during interactions, and to infants’ emotional development in early years.

### The Effect of Exposure to Parental Depression and Anxiety on Infants’ Emotional Development: Future Directions

Our review on the associations between exposure to parents’ emotional expressions and infants’ emotional development in the first year of life reveals the paucity of longitudinal research that addressed the hypothesized links between exposure to maternal and paternal negative emotion, emotional development in infancy, and later psychological outcomes in the offspring. A better understanding of how environmental influences such as exposure to parents’ depression and anxiety contribute to maladaptive outcomes (alone and in interaction with other non-environmental influences) crucially depends on future longitudinal designs that will include assessment of these components from prenatal years to childhood/adolescence in the offspring of depressed/anxious (vs. control) parents.

Recent years were marked by a growing interest in understanding behavioral and neural markers of psychopathology in infancy. Several ongoing longitudinal projects are currently underway to investigate the longitudinal links between observed maternal and paternal emotional expressions/reactions in everyday situations in infancy to later outcomes in childhood (see work the social development study by Bögels and colleagues; e.g., Aktar et al. 2013, 2014; Nikolić et al. 2016) and in adulthood (see longitudinal work by Murray and colleagues on the intergenerational transmission of depression, and social anxiety (e.g., Murray et al. 2007, 2008; Prenoveau et al. 2017) years. Another ongoing project (by Pérez-Edgar and colleagues) addresses the links between infants’ temperamental negative affect, attention biases (obtained via behavioral and physiological measurements) and psychopathology in the first two years of life. The findings from these studies will shed light on the developmental pathways to psychopathology in children of depressed/anxious parents.

Based on our review, we suggest that the following specific issues should be given priority in future research. First, it is important to notice that previous investigations of exposure to parental emotion effects in daily interactions, and in infants’ attention did not include measurements of purely genetic and biological influences. It is therefore unclear to what extent the associations in parents’ and infants’ expressions of emotions and reactions to novel stimuli are explained by environmental exposure after controlling for genetic and biological vulnerabilities in the offspring of parents with depression and anxiety. The studies investigating the links between overall exposure to parents’ depression/anxiety and infants’ attention have relied on mothers’ report. Observations of parents’ negative emotions constitute a more objective index of infants’ actual exposure and therefore must be considered in future studies. Second, exposure effects have not been investigated longitudinally or cross-sectionally in the first year across the developmental transitions signaling the onset of dyadic parent–infant and triadic parent–infant–object associations. For example, we know that mothers with depression are less positive and more neutral/flat during parent–infant interactions in the first half year of life, but we do not know whether these parents also express less positive emotion during infants’ exploration of objects in triadic parent–infant–object interactions in the second half year of life. Future longitudinal investigations of the exposure effects should take into account the milestones in infants’ emotional development. The third issue concerns the associations between behavioral and physiological correlates of infants’ emotional reactivity and of attention allocation. Future research should aim at elucidating the links between infants’ emotional expressions, behavioral reactions and attention to emotion for a more complete picture of infants’ emotional development at different levels of analysis. The final issue is the inclusion of fathers. Previous evidence reviewed above on exposure effects in typically developing infants and in infants of depressed and/or anxious parents predominantly comes from mothers. For a more complete understanding of the exposure effects in the family contexts in early years, it remains essential to investigate differential and interactive effects of exposure to mothers’ and fathers’ negative moods in everyday interactions on infants’ emotional development. Inclusion of both parents is important to understand the compensating or exacerbating effect that the ‘secondary’ caregiver can have in case of a depressed and/or anxiety disordered mother.

### Transactions Between Exposure Effects and Other Mechanisms in the Intergenerational Transmission of Depression and Anxiety: Future Directions

How does exposure to parental depression and anxiety in the first postnatal year interact with inherited genetic/biological predispositions, and other environmental mechanisms to determine adaptive/maladaptive outcomes in the offspring? Inherited genetic markers for depression and anxiety and innate dysfunction in early neuro-regulatory systems in the offspring of prenatally depressed and anxious parents (Goodman and Gotlib 1999) may create additional vulnerabilities, and strengthen the effect of the deviations related of exposure effects on later psychopathology. For example, both depression and anxiety disorders in mothers show high continuity between the prenatal and postnatal years, infants of depressed mothers are therefore likely to have already been exposed to mothers’ depression prenatally (Heron et al. 2004). Newborns of prenatally depressed mothers show altered biochemical/physiological responses (e.g., lower levels of dopamine, higher levels of cortisol levels, and lower vagal tone) that resemble their mothers’ responses (Field et al. 2006). The observed effects of exposure may be partially explained by these biochemical/physiological alterations. Alternatively, the link between exposure to parents’ negative emotions and later psychopathology may be stronger in children of prenatally depressed mothers due to these early vulnerabilities. Likewise, other environmental mechanisms that are at work during and after the first year of life including low marital quality/satisfaction, parenting stress, and socioeconomic disadvantages may diminish/exacerbate exposure effects.

It is therefore important to incorporate other environmental, biological and genetic mechanisms that operate in intrapersonal and interpersonal levels before, during and after infancy together with exposure to parental emotional expressions in future longitudinal studies on early intergenerational transmission of depression and anxiety disorders. Considering the diversity of outcomes in the offspring of anxious and depressed parents, parallel longitudinal measurements of other environmental and genetic factors, along with exposure effects, are crucial in delineating the effects of environmental exposure on concurrent socio-emotional development and subsequent development of depression and anxiety. Family, twin and adoption studies may provide important insights to how exposure-related vulnerabilities in infancy cross-sectionally and longitudinally interact with other genetic or environmental vulnerabilities to determine offsprings’ emotional development and psychopathology. This requires a shift from reliance on self-report measures of psychopathology to the integration of different methodologies allowing the assessment of environmental, biological and genetic mechanisms in future studies addressing intergenerational transmission of depression and anxiety.

### Clinical Implications

The findings reviewed above on the links of exposure to parental depression and/or anxiety on infants’ emotional development highlight the importance of interventions targeting mood and anxiety disorders in the first year of parenthood to decrease the early risk posed by exposure to parents’ negative moods. Although interventions targeting maternal depression are efficient in decreasing parents’ experience of negative emotions (like stress), it remains unclear if they also improve the expression and regulation of negative affect in early parent–infant interactions (Forman et al. 2007). Studies testing the effects of additional interventions targeting parent–infant interactions in addition to mother’s depressive symptoms revealed promising results, although the outcomes were related to other aspects of dyadic functioning (like parental responsivity) than emotional expressions (see Poobalan et al. 2007). Thus, it remains to be investigated whether the interventions targeting expression and regulation of emotion in parent–infant dyads can reestablish the typical emotional expression patterns in early parent–infant interactions. Similarly, relationship-focused (e.g., Promoting First Relationships, Kelly et al. 2008), attachment-based (Bakermans-Kranenburg et al. 2003) and mindfulness-based (Potharst et al. 2017) approaches in early intervention have revealed promising improvements in parents’ functioning and in child behavior (e.g., responsitivity and contingency), while it remains to be tested whether they are also beneficial in the case of parental depression and/or anxiety. It is important that these interventions are compared in future RCT designs that incorporate the measurement of emotional expressions, as well as emotion regulation in diagnosed parents and their infants in the early postnatal years.

## Conclusions

The first major conclusion of the current review is that the extent to which parents express negative emotions with their infants has a direct link to infants’ expressions of emotion in their early interactions with parents. Infants’ emotional expressions echo their parents’ expression of emotion in early interactions. Thus, infants’ repeated exposure to clinically depressed parents’ flat and negative interaction styles in dyadic parent–infant interactions may contribute to the transmission of a similar depressed interaction style from parents to children in the first year of life and constitute risk for the development of depression. Likewise, repeated exposure to fearful and anxious interaction styles from parents with anxiety disorders in triadic parent–infant–object interactions may contribute to infants’ learning of fear and contribute risk for early intergenerational transmission of anxious reactivity patterns from anxious parents to offspring, and to the development of child anxiety.

The second conclusion is that the extent of exposure to parents’ positive and negative emotions is indirectly linked to infants’ attention allocation to others’ positive and negative expressions. Increased exposure to a certain emotion from the parent in the early months seems to be related to less attention to that emotion both in typically developing infants and in infants of depressed or anxious parents. Enhanced attention to positive expression in infants of depressed parents, and the decreased interest to high-intensity negative expressions in infants of mothers with social anxiety disorder may be the result of a protective mechanism that enhances the chances of positively interacting with others, and reduces the chances of exposure to mothers’ faces, and to negative emotion. In turn, enhanced attention to parents’ and others’ negative emotions in infants of depressed and/or anxious parents may be putting certain children at risk for later psychopathology.

## Footnotes

1 The relevant studies were inspected via Google Scholar: The search terms ‘parental depression’ and ‘parental anxiety’ were used in combination with key words ‘interaction,’ ‘infancy,’ ‘emotion’ and ‘face-to-face’ for the first section, in combination with key words ‘infancy’ and ‘emotion’ ‘social referencing’ for the second section, and with key words ‘attention,’ ‘infancy’ and ‘emotion’ for the third section. Other potentially relevant studies were searched through the references of the relevant studies obtained from this initial search.
